# Anna Karenina and the subgingival microbiome associated with periodontitis

**DOI:** 10.1186/s40168-021-01056-3

**Published:** 2021-04-30

**Authors:** Khaled Altabtbaei, Pooja Maney, Sukirth M. Ganesan, Shareef M. Dabdoub, Haikady N. Nagaraja, Purnima S. Kumar

**Affiliations:** 1 Division of Periodontology, College of Dentistry, The Ohio State University, 3180 Postle Hall, 305 W 12th Avenue, Columbus, OH 43210 USA; 2 Present address: Faculty of Medicine & Dentistry, University of Alberta, 5-508 Edmonton Clinic Health Academy, Edmonton, Canada; 3 Department of Periodontics, Louisiana State University School of Dentistry, 1100 Florida Ave., Rm. 3111, New Orleans, LA 70119 USA; 4 Present address: Department of Periodontics, The University of Iowa School of Dentistry, 311 Dental Science Building N, Iowa City, IA 52242-1010 USA; 5 College of Public Health, The Ohio State University, 400-C Cunz Hall, 1841 Neil Ave., Columbus, OH 43210 USA; 6 Division of Periodontology, College of Dentistry, James Cancer Institute, The Ohio State University, 4111 Postle Hall, 305 W 12th Avenue, Columbus, OH 43210 USA

**Keywords:** Microbiome, Chronic periodontitis, Generalized aggressive periodontitis, Comparative metagenomics, Localized aggressive periodontitis

## Abstract

**Background:**

Although localized aggressive periodontitis (LAP), generalized aggressive periodontitis (GAP), and chronic periodontitis (CP) are microbially driven diseases, our inability to separate disease-specific associations from those common to all three forms of periodontitis has hampered biomarker discovery. Therefore, we aimed to map the genomic content of, and the biological pathways encoded by, the microbiomes associated with these clinical phenotypes. We also estimated the extent to which these biomes are governed by the Anna Karenina principle (AKP), which states that eubiotic communities are similar between individuals while disease-associated communities are highly individualized.

**Methods:**

We collected subgingival plaque from 25 periodontally healthy individuals and diseased sites of 59 subjects with stage 3 periodontitis and used shotgun metagenomics to characterize the aggregate of bacterial genes.

**Results:**

Beta-dispersion metrics demonstrated that AKP was most evident in CP, followed by GAP and LAP. We discovered broad dysbiotic signatures spanning the three phenotypes, with over-representation of pathways that facilitate life in an oxygen-poor, protein- and heme-rich, pro-oxidant environment and enhance capacity for attachment and biofilm formation. Phenotype-specific indicators were more readily evident in LAP microbiome than GAP or CP. Genes that enable acetate-scavenging lifestyle, utilization of alternative nutritional sources, oxidative and nitrosative stress responses, and siderophore production were unique to LAP. An attenuation of virulence-related functionalities and stress response from LAP to GAP to CP was apparent. We also discovered that clinical phenotypes of disease resolved variance in the microbiome with greater clarity than the newly established grading system. Importantly, we observed that one third of the metagenome of LAP is unique to this phenotype while GAP shares significant functional and taxonomic features with both LAP and CP, suggesting either attenuation of an aggressive disease or an early-onset chronic disease.

**Conclusion:**

Within the limitations of a small sample size and a cross-sectional study design, the distinctive features of the microbiomes associated with LAP and CP strongly persuade us that these are discrete disease entities, while calling into question whether GAP is a separate disease, or an artifact induced by cross-sectional study designs. Further studies on phenotype-specific microbial genes are warranted to explicate their role in disease etiology.

Video Abstract

**Supplementary Information:**

The online version contains supplementary material available at 10.1186/s40168-021-01056-3.

## Introduction

Periodontitis, an infection-mediated disease that destroys tooth-supporting structures, is the sixth most prevalent disease in the world, affecting over 700 million adults worldwide [1]. The consequences of untreated disease are tooth loss, poor nutritional status, loss of speech, and masticatory function. With the annual cost of periodontal treatment exceeding 15 billion dollars in the USA alone, this disease poses a significant health burden that is comparable to outpatient treatment of cardiac and metabolic diseases [2–4]. Additionally, emerging evidence implicates periodontitis in the pathogenetic pathways of several potentially life-threatening diseases including coronary heart disease, pre-term births, diabetes, and cerebrovascular accidents [5], and therefore, the consequences of untreated periodontitis extend beyond the oral cavity.

Three clinical phenotypes have been most commonly described based on the rate of progression and the pattern of disease within the dentition: chronic periodontitis (CP), localized aggressive periodontitis (LAP), and generalized aggressive periodontitis (GAP) [6]. Although it is established that all three phenotypes have a microbially driven etiology, our current understanding of the microbiota associated with these phenotypes is not sufficient to explain the clinical differences. For example, the prevailing paradigm that similar bacteria are found in CP and GAP [7] does not explain the rapid disease progression seen in GAP, nor does the presence of specific bacteria in GAP and LAP [8–10] explain the involvement of specific teeth in LAP.

Our knowledge of the microbiome associated with chronic and aggressive periodontitis is largely based on phylogenetic characterizations of subgingival communities or investigations of specific bacteria (notably, *Porphyromonas gingivalis*, *Treponema denticola*, and *Tannerella forsythia*, (popularly known as the “red complex” bacteria) and *Aggregatibacter actinomycetemcomitans*) within these ecosystems [10–12]. However, these individual species are part of complex communities and their role in disease causation or perpetuation can be fully understood only when studied in an ecological context. While phylogenetic approaches such as 16S sequencing place these organisms in an ecological framework [13–15], their functional roles can only be inferred. Moreover, most studies are comparisons of only one disease phenotype to healthy controls [7, 16], limiting our ability to separate phenotype-specific associations from those that are common to all clinical presentations of periodontitis. It is not surprising that we have not been able to use microbiomics to develop a robust understanding of how the microbiome contributes to a specific disease phenotype.

High-throughput whole genome sequencing has provided us with an unprecedented view of the genetic composition and functional behaviors of complex biomes that can never be fully characterized by cultivation alone [17]. For example, metagenomic studies of the gut microbiome have revealed that eubiotic communities are similar between individuals but respond in a stochastic or random manner to stresses, resulting in dysbiotic communities that vary from person to person. This is called the Anna Karenina principle (AKP) after the opening line from Tolstoy’s book: “All happy families look alike; each unhappy family is unhappy in its own way” [18]. In the present study, we aimed to investigate what the microbiome is capable of doing and how this functional capacity relates to periodontal health status, in order to develop testable hypotheses about the role of the subgingival microbiome in maintaining health and causing disease. Here, we present the first functional catalog of the subgingival microbiome in the three most common phenotypes of periodontitis. Using a metagenomic approach to characterize the aggregate of bacterial genes in the subgingival microenvironment, we also highlight how each differs from periodontal health. As a tertiary aim, we examined the 1999 (based on disease phenotype) and the 2017 (based on disease extent, severity, and risk) classifications of periodontitis [6, 19] in the context of the subgingival microbiome.

## Methods

### Study population

This study was approved by the institutional review boards of The Ohio State University and Louisiana State University (OSU IRB 2014H0020, LSUHS-NO 8796). Thirty-four nonsmoking, normoglycemic individuals with stage 3 periodontitis as defined by the 2017 classification [19] were recruited and informed consent or assent with parental approval was obtained as appropriate. Additionally, sequences from 25 stage 3 periodontitis and 25 periodontally healthy controls from a previous study [20] were reanalyzed. Periodontal health was defined as clinical attachment loss (CAL) ≤ 1 mm, probing pocket depths (PD) ≤ 3 mm, and mean gingival index < 1. Periodontitis was classified based on both phenotype [6] and disease stage and grade [19] for comparison. Stage 3 grade A (S3gA) was defined as bone loss to age ratio (BL/age) < 0.25, mean plaque index (PI) > 1.5, and mean gingival index (GI) > 1.5; stage 3 grade B (S3gB) as 0.25 < BL/age < 1, PI > 1.5, and GI > 1.5; and stage 3 grade C (S3gC) BL/age > 1, PI ≤ 1, and GI ≤ 1. Chronic periodontitis (CP) was defined as interproximal attachment loss affecting at least 30% of the sites in individuals between 40 and 80 years of age. CP was diagnosed based on at least two periodontal assessments over at least 2 years. Localized aggressive periodontitis (LAP) was defined as interproximal attachment loss affecting first molars and incisors (and no more than 2 other teeth) in systemically healthy individuals below 40 years of age. An additional requirement was a contributory family history (of early tooth loss). Generalized aggressive periodontitis (GAP) was defined as rapid progressing interproximal attachment loss in systemically healthy individuals below 40 years of age that did not follow the patterns of distribution in the dentition described for LAP. Exclusion criteria included age below 8 years, current pregnancy, requirement for antibiotic prophylaxis prior to dental therapy, HIV infection, long-term (greater than 3 months) use of medications known to cause gingival changes (e.g., immunosuppressants, phenytoin, calcium channel blockers, aspirin, NSAIDS, bisphosphonates, or steroids), antibiotic therapy within 3 months of sample collection, and history of previous subgingival periodontal therapy.

Sample size was estimated based on the probability of least an 80% chance of detecting individual genes that differed in abundance by at least 0.01% between any two groups.

### Clinical procedure

Patients were recruited after an initial screening and diagnosis visit when periodontal health related metrics (clinical attachment loss (CAL), probe depths (PD), bleeding on probing (BOP), gingival index (GI), and plaque index (PI)) were recorded. Since a common clinical presentation in all three disease phenotypes is that only some sites are affected by disease, 3 non-contiguous sites with clinical attachment loss (CAL) > 5 mm and probing depth (PD) ≥ 6 mm on 3 different teeth were selected in subjects with periodontitis. Samples were collected 1 week following the initial screening visit. This strategy was adopted to minimize the effects of bacterial translocation due to periodontal probing. Samples collected by inserting sterile endodontic paper points (Caulk-Dentsply, Milford, DE, USA) to the depth of the periodontal pocket for 30 s. From periodontally healthy subjects, samples were similarly collected and pooled from 15 non-contiguous interproximal sites. Paper points were immediately placed in 100 μL of RNA*Later*, temporarily stored in ice, and were transferred to − 20 °C until analysis.

### DNA isolation and sequencing

Bacterial DNA was isolated from paper points, using Qiagen DNA MiniAmp kit (Qiagen, Valencia, CA, USA) and quantified using Qubit fluorometer. Fifty nanograms of DNA was used to generate libraries with an Illumina TruSeq kit according to the manufacturer’s instructions. Quantified and pooled libraries were clustered on the Illumina HiSeq 4000 system (Illumina, Inc., San Diego, CA, USA) and 150 bp paired-end sequencing performed. Sequences for all 59 samples are deposited in the Sequence Read Archives under the project ID PRJNA552294 and PRJNA508385.

### Statistical analysis

Trimmed and filtered sequences were uploaded to the MG-RAST metagenomics analysis pipeline (version 3.3.6) [21, 22] (Argonne National Laboratory) for quality processing and basic functional analysis. The MG-RAST API [23] and the custom Python library we developed to access it and analyze/visualize results were used throughout the analysis process to download relevant data and pipeline results (available for download at http://github.com/smdabdoub/PyMGRAST).

The phylogenetic profile of each group was determined using Kraken v1.1 [24] with a database constructed from a list of complete genomes from the Human Oral Microbiome Database [HOMD], as of September 19, 2017 (GenBank IDs available in Supplement). Although Kraken provides data at all levels of taxonomy, analysis was performed at the genus and species levels, showing high levels of specificity, accuracy, and coverage. Only taxa present in at least 20% of subjects in each group and constituting ≥ 0.000005% of the relative abundance were retained. Annotations with Kraken were done using the Ohio Supercomputer facilities (www.osc.edu). Knowledge-based assignments were made for gram staining and oxygen tolerance characteristics of the OTUs (Python script available on https://github.com/akshayparopkari/kadambari). As-yet-uncultivated phylotypes were annotated based on the characteristics of their cultivated phylogenetic neighbors. The taxonomic attribution of each function was resolved by matching the unique ID of each annotated sequence using the SQLite3 database (Version 3.28.0). Briefly, functionally annotated sequences were matched with the primary output of Kraken using SQLite3. The full SEED functional ontology of these sequences was obtained by matching their accession IDs to a reference database of the SEED subsystem.

Species diversity and richness were interrogated using the Chao 1 diversity index and abundance coverage estimator (ACE), and group-wise significance interrogated with rank-based nonparametric tests (Wilcoxon rank-sum test and Kruskall-Wallis test (using PhyloToAST diversity.py [25])). Dissimilarity between samples was calculated using Bray-Curtis metrics to estimate beta diversity. Nonmetric multidimensional scaling (NMDS) of Bray-Curtis distances was performed using the R package *Vegan* [24], and the R package *Plotly* used for visualization [26]. Significance of clustering was interrogated using *adonis* function, (QIIME implementation of *vegan* package in R) with 999 permutations. Similarity percentage analysis (SIMPER) of Bray-Curtis distances was used to determine the drivers of separation [27].

*k*-means clustering was used to estimate the variance in the disease-associated microbiome. Bray-Curtis dissimilarity (computed from the relative abundances of genes and species in subjects with disease) was used as input and silhouette width used to estimate the number of clusters [27]. A silhouette was created for each cluster based on the closeness of the members and separation from others. The silhouettes were then combined onto a single plot, providing an evaluation of the validity of the number of clusters. Factors that contributed to variance in the microbiome were identified using a between-class analysis method. Briefly, relative abundances of genes and species in subjects with disease were input into a principal coordinate analysis, and the eigenvectors that explained 75% of the variance in the microbiome were inducted into a linear discriminant analysis. Linear discriminant analysis (LDA) for dimensionality reduction of CSS-normalized s-OTU counts was performed using scikit-learn v0.18.0 [28] and plots were visualized using PhyloToAST. MANOVA/Wilks lambda was used to test for significance of LDA clustering.

Between-group differences in abundances and prevalence were estimated for both phylogenetic and functional data. Statistical testing for differences in relative abundances was performed using the DESeq2 package with Bayesian shrinkage of estimators (R package apeglm) [29, 30]. *p*-values were adjusted for multiple testing (FDR < 0.1, FDR-adjusted Wald test). Barycentric plots based on the functions that passed DESeq2 adjustments were graphed using the *ggtern* package in R [31]. Comparisons of functional potential between groups were made in the context of the KEGG (Kyoto Encyclopedia of Genes and Genomes) [32] and the SEED [33] ontological hierarchies. Presence/absence of the features was interrogated using Fisher’s exact test and odds ratios calculated with the R packages *Questionr* and *DescTools*.

Core features were calculated based on presence of the feature in at least 80% of patients with the particular condition. The SparCC package in python was used to construct network dependencies between features [34]. To decrease the occurrence of spurious associations due to rare taxa, co-occurrence networks were computed only on the core taxa [35]. Correlation was estimated on log-transformed abundances of the core features, and *p*-values computed following 99 bootstraps. Significant co-occurrences (rho ≥ 0.6 and *p* < 0.05) were used to create the graph structures. Gephi v0.9.1 [36] was used to visualize the resultant networks. The Zi-Pi plot calculations were done using the formula of Guimerà and Nunes Amaral and graphed using the simplified method of Olesen [37, 38].

The ability of genes to discriminate between groups was examined using a machine-learning algorithm (RandomForest package in R). Two thirds of the dataset was used to train the classifier, which was tested on the remaining data (1000 trees/10-fold cross validation). For all iterations of the test a “confusion table” was created for each of the exposures based on the number of correctly classified and misclassified samples, and this data was used to compute sensitivity and specificity. The robustness of the classifier was evaluated using ROC curves (ROCR package in R).

## Results

Our primary aim was to characterize the functional and phylogenetic profiles of the subgingival microbiome in individuals with periodontitis. To do this, we obtained 72 million classifiable sequences from deep periodontal pockets of 59 systemically healthy subjects with periodontitis, and 31 million sequences from 25 periodontally healthy individuals. All subjects with periodontitis were classified as stage 3 based on disease severity and complexity (Table 1), with 17 patients demonstrating the molar-incisor phenotype (equivalent to LAP phenotype). Four subjects were classified as stage 3 grade A (S3gA), 22 as S3gB, and 33 as S3gC. Twenty-five subjects were classified as chronic periodontitis (CP; age range, 56–61 years), 17 as generalized aggressive periodontitis (GAP; age range, 24–32 years), and 17 as localized aggressive periodontitis (LAP; age range, 15–19 years). Their sequences represented 8336 functionally annotated microbial genes and 454 taxa.

**Table 1:** Clinical and demographic characteristics of the study population

Demographic and clinical criteria	CP (***N*** = 25)	GAP (***N*** = 17)	LAP (***N*** = 17)
Age (range, median)	56–61, 59	24–32, 26	15–19, 17
Gender (% male)	72	52	54
Ethnicity (number of Caucasian to African American to Hispanic to Asian)	17:5:2:1	8:5:3:2	0:17:0:0
% bone loss/age at most affected site (range)	0.73–3.1	0.86–3.57	0.9–2.8
Probe depth at deepest site (range, median)	6–9, 6	5–8, 6	7–10, 7
Number of teeth with furcation involvement (range, median)	0–6, 2	1–3, 2	1–2, 2
Clinical attachment loss at deepest site (range, median)	7–10, 7	6–9, 7	7–10, 8
Bleeding on probing (% sites) (range, median)	20–100, 80	20–80, 65	10–40,
Molar-Incisor pattern (number of subjects)	0	0	17
Mean plaque index (Loe and Silness) (range)	1.2–2.3	1.1–2.1	0.5–0.7
Mean gingival index (Loe and Silness) (range)	1.3–2.5	0.9–2.2	0.6–0.9
Number of individuals with tooth loss	6	3	3
Number of individuals with known history of tooth loss due to periodontitis	5	2	3
Number of teeth lost/individual (excluding third molars, orthodontic reasons and congenitally missing) (range)	0–5	0–3	0–1
Number of teeth known to be lost to periodontitis/individual (range)	0–2	0–3	1

### Subgingival microbiomes follow the Anna Karenina principle

We began our analysis by creating a catalog of disease- and health-associated genes. In all three disease phenotypes, 73.8% of 454 taxa and 60% of 8336 genes were identified in 2 or more individuals within each group. Disease-associated microbiomes demonstrated greater beta-dispersion [39], with only 47% of disease-associated metagenome being shared by 80% or more of individuals with periodontitis (common core metagenome). On the other hand, over 73% of genes were identified in the common core of periodontal health, indicating that periodontitis-associated microbiomes follow the Anna Karenina principle (AKP). However, AKP was most evident in chronic periodontitis (34% of transcripts contributing to common core metagenome), followed by generalized aggressive periodontitis (51%) and localized aggressive periodontitis (62%).

**Fig. 1:**
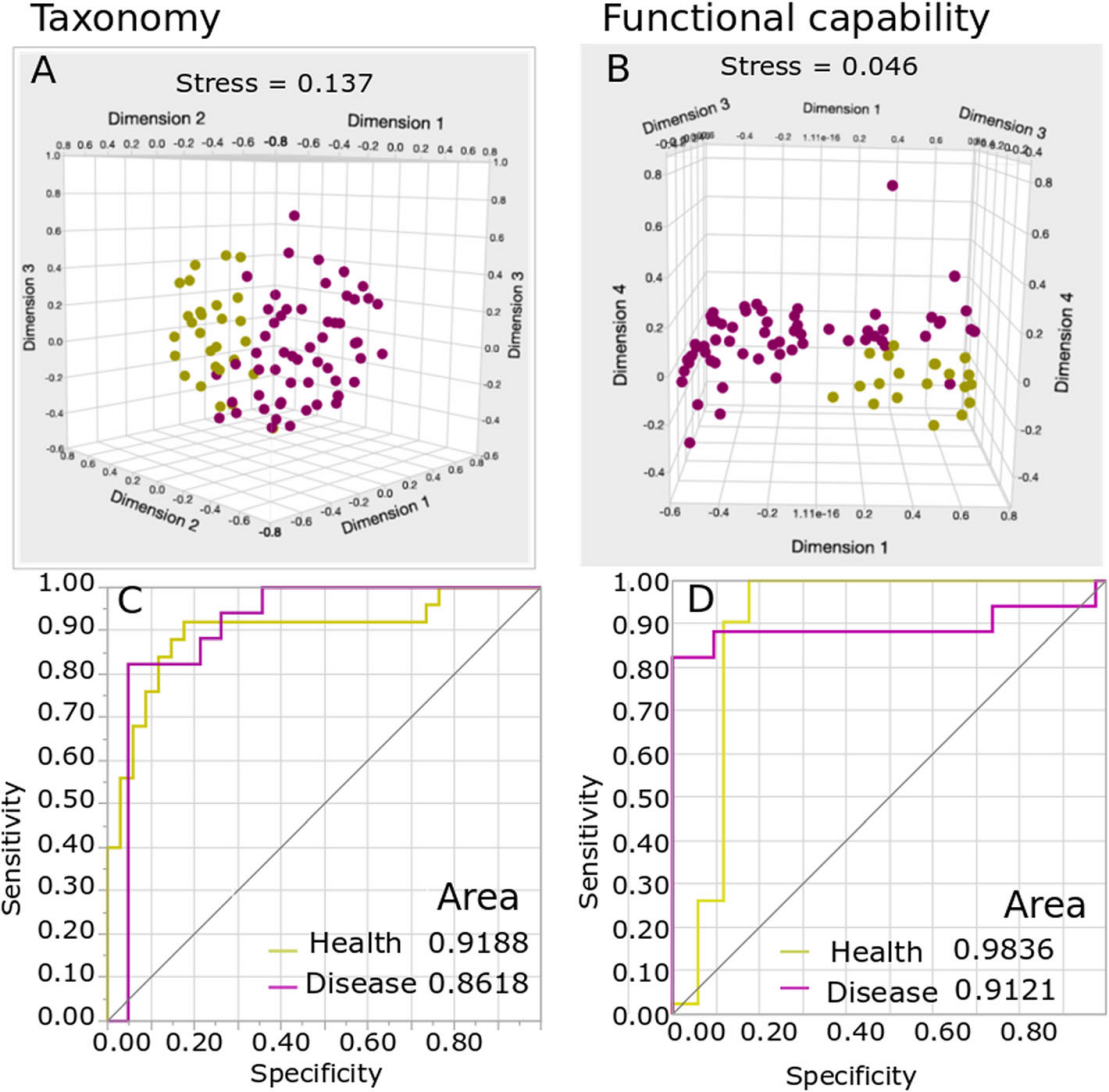
Between-class analysis of phylogenetic and functional profiles in health and disease. Nonmetric multidimensional scaling (NMDS) and receiver operating characteristic (ROC) curves shown. The first three dimensions of species-level (**a**) and gene-level (**b**) Bray-Curtis distances are shown (*p*-value < 0.001 for **a** and **b**). Each purple circle represents one of 59 subjects with periodontitis and each yellow circle represents one of 25 periodontally healthy individuals. The ability of disease-specific and health-specific indicators to predict each state is shown in **c** (phylogenetic metrics) and **d** (functional metrics)

We then queried whether AKP would preclude robust differentiation between health and disease. To do this, we computed a “consensus disease profile” based on genes and taxa found in all 59 patients and compared this to health using nonmetric multidimensional scaling of Bray-Curtis pairwise dissimilarities. The difference between health and disease was significantly greater than differences between any two individuals with disease (Fig. 1a, b, *p* < 0.001, PERMANOVA). A Random Forest Classifier (RandomForest package in R) classifier was able to predict disease with 87% sensitivity and 91% specificity based on functional profiles and with 72% sensitivity and 78% specificity when using phylogenetic metrics (Fig. 1c, d). Overall, 28% of genes were uniquely observed in disease, and 12% were unique to health, while 26% exhibited significant differential abundances in health and disease.

### Disease phenotype explains microbiome variance better than disease grade

**Fig. 2:**
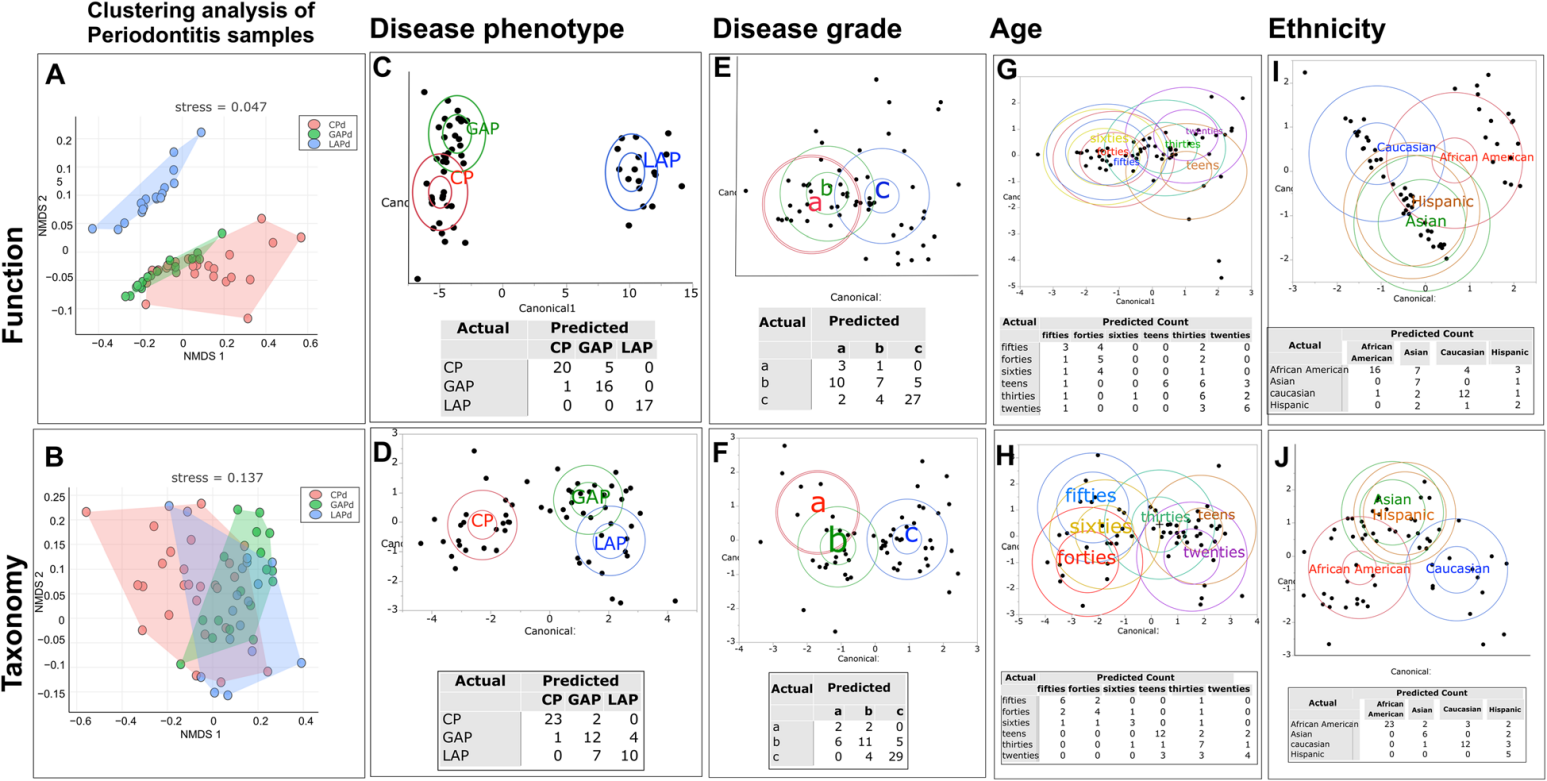
Factors that explain variance in the subgingival microbiome. *k*-means clustering of Bray-Curtis distances revealed three clusters (LAP blue, CP red, GAP green) based on function (**a**, *p*-value < 0.001) and taxa (**b**, *p*-value < 0.001). The ellipses represent the centroids of each cluster. Linear discriminant analysis of relative abundances of function (top panels) and phylogeny (bottom panels) revealed that disease phenotype (**c**, **d**), but not disease grade (**e**, **f**), was able to discriminate between subjects with disease. The differences based on age-decade (**g**, **h**) and ethnicity (**i**, **j**) were explained by the disease phenotype. In each cluster, the larger ellipse indicates the 95% confidence region to contain the true mean for the group, and the smaller (inner) ellipse represents the region estimated to contain 50% of the population for the group. The misclassification rates of each variable are shown within each panel

Having established that periodontitis differed significantly from health both taxonomically and functionally, we next investigated if differences could be discerned within the periodontitis-associated microbiome using unsupervised cluster analysis (*k*-means clustering). Bray-Curtis dissimilarity distances (computed from the relative abundances of genes and species in subjects with disease) were used as input and silhouette width used to estimate number of clusters. We identified three distinct clusters taxonomically and functionally (*p* = 0.0008 and 0.001 respectively, ADONIS test of Bray-Curtis Dissimilarity Index, Fig. 2a, b). We then investigated the factors that drove these differences using a between-class analysis method that combines principal coordinates analysis with linear discriminant analysis (Fig. 2c–j). Relative abundances of genes and species in subjects with disease were used as input. Disease phenotype, ethnicity, and age emerged as the strongest drivers of clustering. Disease phenotype yielded the lowest degree of misclassification both taxonomically and functionally while significant misclassification was evident when using disease grade as a discriminant. Furthermore, disease phenotype explained the strong ethnicity and age-based clustering, since most of the younger individuals and those of African American ethnicity belonged to the LAP group (Fig. 2i).

### Same players, different teams

Since targeted microbial investigations have previously suggested that localized aggressive periodontitis has a distinct microbial profile while the microbiota of chronic and generalized aggressive periodontitis are similar [40], we tested the hypothesis that GAP and CP are microbially similar while LAP is a taxonomically distinct entity. NMDS revealed significant class separation between the 3 diseases (*p* < 0.001, PERMANOVA of Bray-Curtis Dissimilarity Index, Fig. 2b). We then investigated whether GAP and CP are microbially more similar than GAP and LAP by computing pairwise dissimilarities (Bray-Curtis) between each GAP and CP subject, as well as each GAP and LAP subject. Taxonomically, GAP was more similar to LAP than to CP (*p* < 0.001, Dunn’s test for multiple comparisons).

**Fig. 3:**
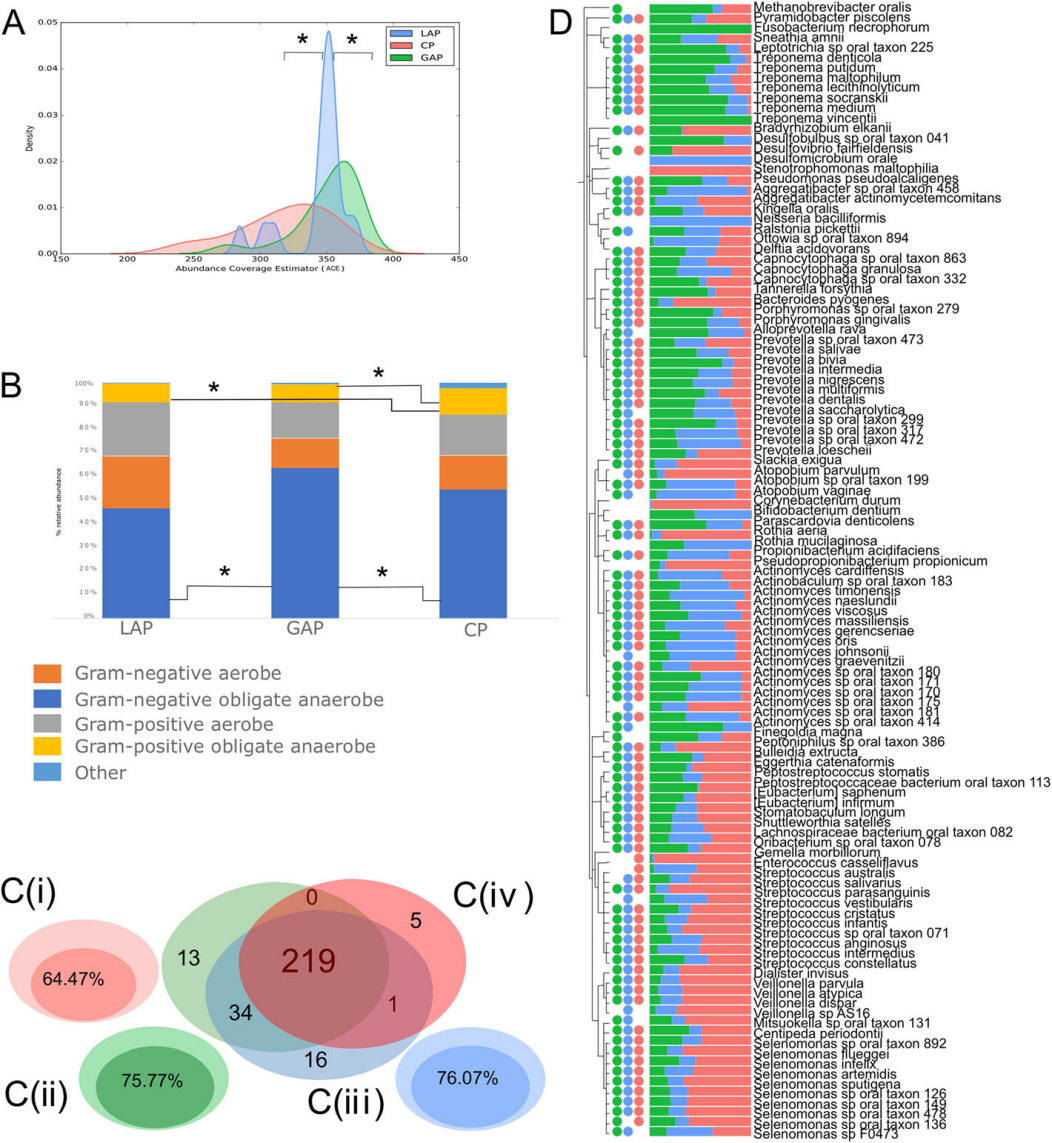
Disease-specific taxonomical indicators. Density curves of alpha diversity (ACE) are shown in **a**. The peak indicates the median values for each group, and the *x*-axis shows the data range. LAP exhibited significantly lower alpha diversity than the other 2 groups (*p* < 0.0001, Dunn’s test). Distribution of species-level taxa by gram staining characteristics and oxygen requirements in is shown in **b**. GAP patients demonstrated significantly greater gram-negative anaerobic bacteria and lower gram-negative aerobic bacteria when compared to the other two groups (*p* < 0.01, Dunn’s test for multiple comparisons). Percent of the microbiome that is shared by 80% or more of individuals (common core microbiome) with CP, GAP, and LAP are graphically indicated by the Euler graphs **c** (i–iii), as well as the number of core species shared by all three diseases is shown in **c** (iv). Phylogenetic tree of species that were significantly different between groups (*p* < 0.05, FDR-adjusted Wald test) are shown in 3D. Bars represent normalized mean relative abundances, while the solid circles indicate species that belong to the common disease core. Data supporting this figure can be found in Supplemental Table 1

Since the NMDS indicated that these three diseases were microbially discrete entities, we examined the taxonomic features that contributed to class separation. LAP exhibited significantly lower species richness (as measured by the ACE and Chao 1 indices) than the other groups (*p* < 0.05, Dunn’s test, Fig. 3a), and the three groups also demonstrated significant differences in beta diversity. While all 3 diseases were dominated by gram-negative anaerobic bacteria, (representing 56.8%, 62.5%, and 47.9% of the abundance in CP, GAP, and LAP, respectively (Fig. 3b)), the abundances of these groups were significantly greater in GAP when compared to LAP (*p* = 0.03, Wilcoxon nonparametric test). By contrast, gram-positive anaerobic bacteria were significantly higher in CP when compared to either GAP or LAP (*p* < 0.04, Wilcoxon).

Interestingly, 349 out of 416 species were identified in all the three diseases, and only 28 species were unique to any one of the three diseases. Collectively, the unique species constituted less than 0.03% of the abundance in each group. Each disease condition had core taxa that constitute more than 50% of the identified taxa in the condition (Fig. 3c (i–iii)). When core taxa of each condition were compared, most species were present in the cores of all 3 conditions (Fig. 3c (iv)). 138 OTUs were found to be significantly differentially abundant between any two disease states (*p* < 0.05, FDR-adjusted Wald test — Fig. 3d and Supplementary Table 1). Using similarity percentages (SIMPER) analysis, we identified OTUs that explained 70% or more of the class separation. Several OTUs which significantly contributed to the separation were also common core taxa, demonstrating that the diseases differ in the ratios of their predominant shared taxa. One hundred and seven OTUs were significant contributors to the separation between GAP and LAP; of these, 65 were part of the common core species of GAP and 63 formed the common core of LAP (Fig. 3d, supplementary Table 1). Similarly, 50 species, 39 of which were members of the core microbiomes of GAP and CP, contributed to the separation between GAP and CP. The separation between CP and GAP was driven by *Aggregatibacter actinomycetemcomitans*, *Fusobacterium nucleatum*, *Treponema socranskii*, and several members of the genera *Actinomyces*, *Campylobacter*, *Prevotella*, and *Capnocytophaga*. The separation between LAP and GAP was mainly driven through *Porphyromonas gingivalis* and members of the genera *Neisseria* and *Actinomyces*.

**Fig. 4:**
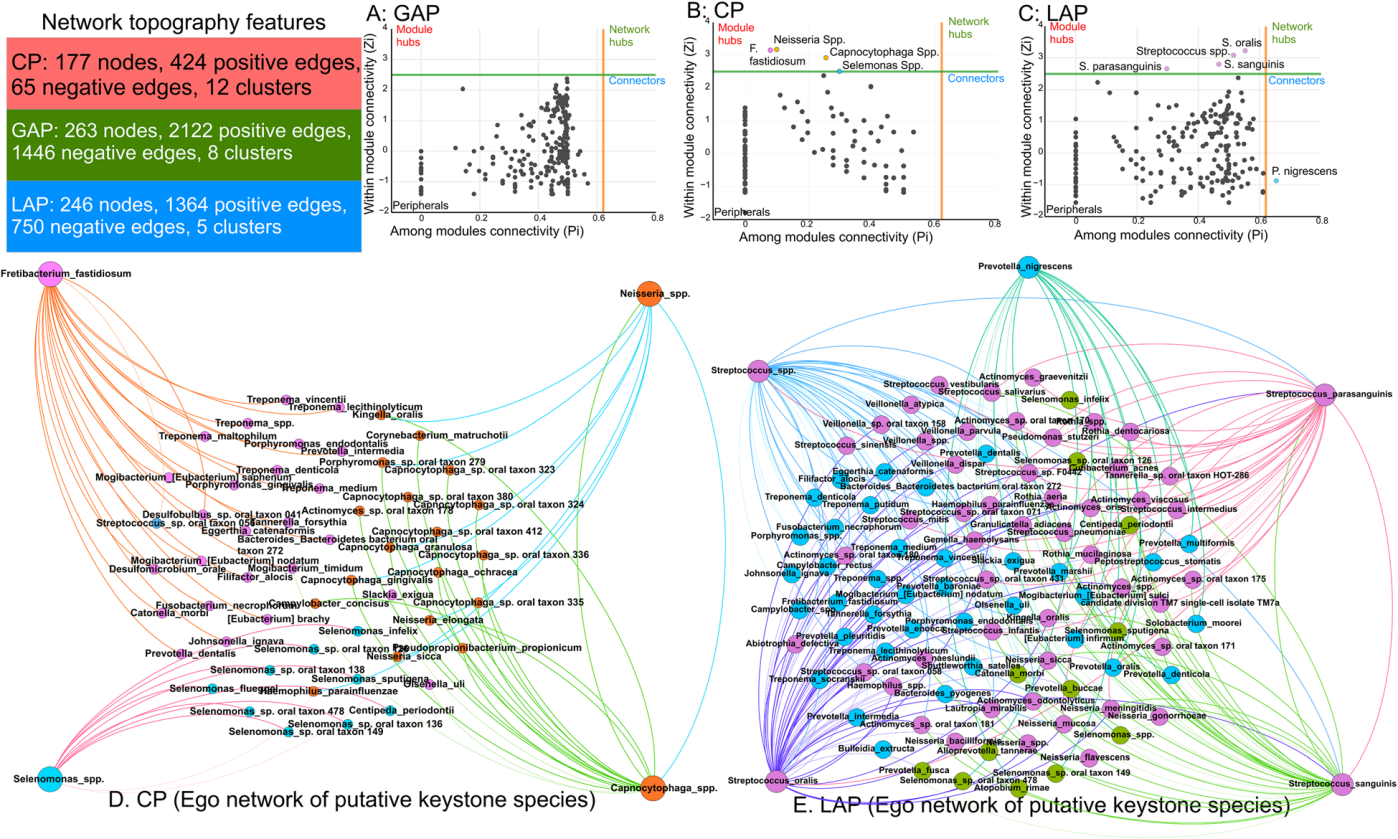
Same players, different teams. Zi-Pi plots of co-occurrence networks in generalized aggressive periodontitis (**a**), chronic periodontitis (**b**), and localized aggressive periodontitis (**c**). The text box illustrates the general topographical characteristics of each network. The plots show nodes with low connectivity (peripherals), and the putative keystone species, which are separated into local/module key players (module hubs), key players across different modules (connectors), and the combination of the two categories (network hubs). Network graphs of chronic periodontitis and localized aggressive periodontitis (**d**, **e**) show the ego network of species that have first-degree edges with the putative keystone species. A similar network graph was not possible for generalized aggressive periodontitis since there no keystone pathogens were identified in the Zi-Pi plot. Nodes are colored based on their modules. Thick edges represent positive correlations, and faint lines represent negative correlations. Data supporting this figure can be found in Supplemental Table 2

Since inter-bacterial interactions play a large role in influencing microbial assemblages, we used graph theoretics to assess connectivity between species. The underlying rationale for this analysis is that taxa with the strongest connections demonstrate superior adaptation to their niche. As a corollary, diseases that present similar microenvironments will demonstrate greater co-dependency between member species than diseases that are different. The network topography is summarized in Supplemental Table 2. While GAP and LAP demonstrated robust hubs with 3568 and 2114 edges, CP presented a sparse topography, with only 489 connections, attesting to its phylogenetically idiosyncratic presentation (Fig. 4). Zi-Pi plots of both CP and LAP demonstrated expansive nodes with several putative keystone species in the network topography, while the node distribution in GAP was equitable (preventing us from creating a Zi-Pi plot) and did not demonstrate any candidate keystone species. Together, the data suggest that patients with GAP and LAP have a more homogeneous subgingival microenvironment than those with CP, which may explain the taxonomic heterogeneity observed in CP. Based on the clinical observation that 35% of untreated cases of LAP progress to GAP [41], we hypothesize that loss of the influential key players found in LAP creates a state of flux that, when observed cross-sectionally, gives rise to the observation that GAP is a distinct disease phenotype. This theory is further supported by observations that individuals with GAP demonstrate low serum antibody response to the microbial constituents, leading to its continuous periodontal destruction [40]. This is unlike the other two phenotypes which can self-arrest with time.

### The LAP microbiome is functionally distinguishable from CP and GAP

**Fig. 5:**
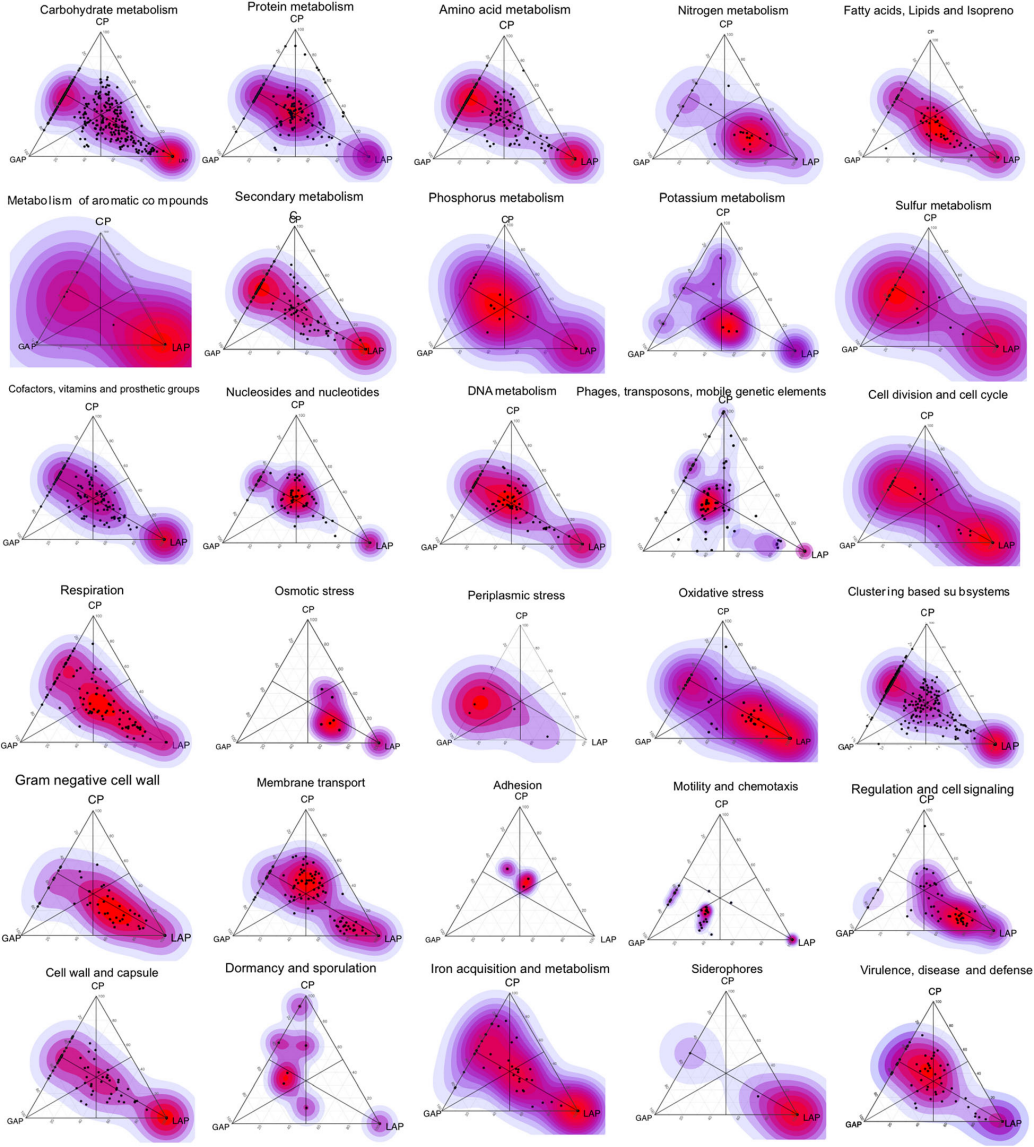
Disease-specific functional indicators. Barycentric plots of significantly different virulence functions in the three groups (*p* < 0.05, FDR adjusted Wald test) are shown. Each dot represents a gene. The three groups generalized aggressive periodontitis, chronic periodontitis, and localized aggressive periodontitis (GAP, CP, and GAP) are used as vertices. Within each plot, the coordinates of each gene are determined by the weighted average of the coordinates of all genes, and the weights are given by the relative abundance of the gene in that group (LAP, GAP, and CP). Data supporting this figure can be found in Supplemental Table 3

Since the three disease phenotypes demonstrated several taxa in common, we tested the hypothesis that there would be significant functional overlap in their respective associated microbiomes using the SEED ontology to annotate genes and the KEGG database for pathway identification. A greater degree of class separation was evident based on functional capabilities than on taxonomic profiles (*p* < 0.0001, PERMANOVA of Bray-Curtis Dissimilarity, Fig. 5a). 20.61% of the LAP metagenome (1278/6200 genes) was unique, in that, these genes were not present in either GAP or CP (Fig. 5b and Supplemental Table 3). Twenty percent of these unique genes did not have functional role assignments, pointing to gaps in our knowledge of the microbiome of localized aggressive periodontitis. Forty percent of unique genes encoded enzymes for anaerobic degradation of aromatic compounds, methanogenesis, lysine and acetyl CoA fermentation, and anaerobic respiratory reductases. Twenty percent of the unique genes coded for gram-negative cell structures and 5% for gram-negative phages. 27.96% (2037/7286) of the LAP metagenome differed significantly from that of GAP (*p* < 0.05, FDR-adjusted Wald test, DESeq2). The LAP microbiome demonstrated greater capacity for inositol catabolism, and Lipid A, lipopolysaccharide, and peptidoglycan biosynthesis when compared to GAP (*p* < 0.05, FDR-adjusted Wald test, DESeq2). Additionally, the LAP biome demonstrated a 4-fold to 144-fold greater enrichment of genes encoding c-type cytochrome and molybdenum cofactor biosynthesis, iron-sulfur clusters, formate dehydrogenase, and oxidative stress response. 27.5% (2019/7340) of the LAP metagenome differed from CP. This was attributable to a higher representation of genes encoding acetyl CoA, lactate, mixed-acid and lysine fermentation, methanogenesis, anaerobic respiratory reductases, dehydrogenases, dehydratases and anaerobic toluene, and ethylbenzene degradation. Also overrepresented in the LAP metagenome were membrane transport functions (type II, III, IV, V, and VI secretions systems and ABC transporters), and functions related to quorum sensing and biofilm formation (Autoinducer-2 transport and processing, biofilm adhesins, and histidine kinase sensors).

### The GAP microbiome — a functional chimera

Pairwise dissimilarity analysis revealed that the GAP metagenome was intermediate between CP and LAP (*p* = 0.91, Dunn’s multiple comparisons test on Bray-Curtis distances between GAP-CP and GAP-LAP). GAP shared 77% of its metagenome with CP and 64% of its metagenome with LAP. To reduce bias induced by sparse data, we next used core genes in each group to compute these distances. Not surprisingly, we observed a lower similarity within the common core metagenomes, but a more balanced difference, with 56% of genes shared by CP and GAP, and 55.1% by GAP and LAP. Since severe attachment loss in young adults in the presence of clinical inflammation and local factors could represent either early-onset chronic periodontitis, a generalized form of the molar-incisor phenotype, or true de novo aggressive periodontitis [41], we examined clustering of the GAP samples alone. NMDS did not reveal significant separation between the 17 samples, suggesting that this chimeric effect cannot be readily attributed to heterogenous diseases.

Fourteen percent of the core genes shared by LAP and GAP encoded as yet unknown functions (Supplemental Table 4). Among the characterized genes, the predominant shared functionalities were related to an anaerobic lifestyle. These included genes encoding heme- and hemin-dependent respiration, dehydrogenases, electron donors and acceptors other than oxygen (namely, nitrate, sulfate, hydrogen, and ferric iron), and fermentation. Other shared functions included polyamine metabolism, flagellar biosynthesis, and gram-negative cell wall components (including peptidoglycan biosynthesis), response to oxidative and osmotic stress, resistance to antibiotics and toxic compounds, phages, and conjugative transposons. The differences in the microbiomes of LAP and GAP were attributable to lower abundances of membrane transport functions (type II, III, IV, V VI, and ABC transporters), quorum sensing and biofilm formation in and higher levels of sporulation and dormancy, phages and transposable elements in GAP. Pathways involved in biofilm stability were also lower in GAP in comparison to LAP, and even lower in CP when compared to GAP.

The functional roles of 19% of the genes shared by CP and GAP were unknown. Both CP and GAP demonstrated a collective capacity for metabolism of amino acid, organic compounds, alcohols, and glycogen. The other shared functionalities included capsule and cell wall synthesis, response to oxidative and osmotic stress, and resistance to antibiotics and toxic compounds, phages, and conjugative transposons. Flagellar components and proteins associated with flagella biosynthesis and assembly machinery are also exclusively enriched in GAP as compared to CP, as were potent inflammatory triggers such as lipopolysaccharides and peptidoglycans. Other abundant functions of GAP include dormancy and sporulation, invasion and intracellular resistance, iron acquisition and siderophores, and multidrug antibiotics efflux pumps.

## Discussion

Culture-based approaches to characterizing the subgingival microbiome spawned a slew of studies on individual bacteria, their responses to environmental shifts, and their roles in disease causation as independent operators [42–46]. However, cultivation independent methods have revealed that polymicrobial diseases are caused by the collective actions and interactions of the microbiome [47, 48], and that the “invisible” members of a microbial community have important contributions to these interactions [49, 50]. When we used a top-down approach to map the genomic content of and the biological pathways encoded by the microbiomes associated with chronic, localized, and generalized aggressive periodontitis, a surprising picture of the three phenotypes emerged.

Healthy microbiomes demonstrated significantly lower beta-dispersion than disease, similar to what has been reported in the gut microbiome [51]. This effect has been called the Anna Karenina principle and suggests that healthy microbiomes operate under stricter host control than do diseased ones. Subjects with periodontitis were carefully selected for clinical homogeneity: all sampled sites demonstrated similar attachment loss, pocket depths, and periodontal inflammation. Therefore, the variance in the microbiome could not be explained by disease severity. This dispersion was more apparent in taxonomy than in the functional profiles, corroborating our earlier finding that the microbiome associated with periodontitis is taxonomically heterogenous but functionally congruent [20]. This was also corroborated by identifying a set of putative periodontal pathogens and perturbed functions common to all three diseases, suggesting that certain genes and taxa are broadly associated with the disease process. This is not surprising, since it has long been recognized that periodontitis is a microbially heterogenous disease [52], this observation was largely based on examination of selected species or meta-taxonomic cross-sectional studies. However, by overlaying robust clinical metrics on the genomic content of the subgingival microbiome, we were able to identify that not all clinical phenotypes of periodontitis are equally heterogenous and that the heterogeneity does not extend to microbial functions. We realize, as we say this, that this is a cross-sectional study, and therefore, it is not designed to identify patterns in microbial community dynamics. Our findings serve to further reinforce the critical need for adequately powered longitudinal studies that combine granular clinical metadata with open-ended explorations of gene expression patterns and protein-protein networks, and rigorous and improved modeling of stochastic events in microbiome assembly.

Another observation was that most functions which discriminated between health and disease were significantly more abundant in disease, suggesting that the disease environment is associated with expanded functionality. Our study reveals that particular functional capability is required for life in the anaerobic, pro-oxidant, heme-rich environment of a pathologically deepened subgingival sulcus. For example, lipopolysaccharide biosynthesis, iron transport, stress response, fermentation, and metabolism of secondary amino acids were uniformly enriched in all three diseases when compared to health, even though some of them were more abundant in one of the three disease phenotypes.

We also found that species demonstrating a fitness for this environment are different in each individual, highlighting challenges associated with developing species-based biomarkers or vaccines for periodontitis. For example, flagellar proteins, potent inflammatory triggers, mapped to a variety of organisms (e.g., species belonging to the genera *Treponema*, *Selenomonas*, *Pseudomonas*, and *Campylobacter*), while genes encoding dormancy and sporulation, invasion and intracellular resistance, iron acquisition and siderophores, multidrug antibiotics efflux pumps were assigned to several species within the genera *Aggregatibacter*, *Porphyromonas*, *Atopobium*, and *Prevotella*, among others.

Integrating data from the three most common phenotypes of periodontitis allowed for identification of phenotype-specific indicators, elucidating their potential role in disease causation. Disease-specific indicators were more readily evident in the LAP microbiome than GAP or CP. When we examined the unique and overrepresented suite of genes in LAP, a picture emerged of a community with greater fitness for an anaerobic, proteolytic lifestyle, and a higher capacity for virulence than CP or GAP. For example, genes that encode the “acetate switch”, permitting a transition from rapid growth to a slower, acetate-scavenging lifestyle [53], as well as dehydrogenases, hydratases, and anaerobic reductases were identified only in LAP. Anaerobic glycerol phosphate-3-hydrogenase genes, which play a critical role in utilizing alternate nutritional sources [54] were consistently higher in LAP when compared to the other two. Genes encoding c-type cytochrome and molybdenum cofactor biosynthesis, iron-sulfur clusters, and formate dehydrogenase were also overrepresented in this cohort. Molybdenum cofactor is essential in bacterial respiration and energy conversion, especially in those species that do not have appreciable plasticity in their metabolic and respiratory pathways [55]. Molybdenum metabolism is also tightly connected to iron-sulfur cluster synthesis [56]. Formate is not only an important byproduct of anaerobic respiration, it is also a substrate for many sulfate reducing bacteria, for example, *Campylobacter* and *Prevotella* [57]. Its synthesis is mediated by formate dehydrogenase, an enzyme that contains molybdenum cofactor and iron-sulfur clusters. C-type cytochromes regulate several key pathogenic processes, notably heme synthesis, oxidative stress response, nitrosative stress response, and siderophore production [58]. This small set of genes that were unique to LAP may provide insights into disease etiology and development of LAP-specific microbial biomarkers and deserve further investigation.

On the other hand, the microbiome of GAP shared important taxonomic and functional features with both LAP and CP. This “chimera”-like appearance might, in part, explain the clinical observation that 35% of untreated cases of LAP progress to GAP [41]. Furthermore, the absence of the putative keystone species in GAP could indicate an ecosystem in a state of flux, which can explain the poor immune response from the patients, and the lack of the ability of the disease to self-arrest [59]. While the absence of clustering within the GAP samples argues against this, it is possible that our small sample size precluded a statistically significant clustering. Our observations lead us to question whether GAP is indeed a unique disease or represents, for some individuals at least, a “halfway house” between LAP and CP.

We noticed progressively greater beta-dispersion and the concomitantly smaller common core microbiomes when moving from LAP to GAP to CP, pointing to greater personalization of the disease-associated microbiome in older individuals with CP (CP average age in this cohort 58 ± 2 years old). Two possible explanations present themselves: one is that the microbiome naturally shifts with increasing age; and the age of the microbiome when dysbiosis sets in could determine the disease-associated profile. The other is that personalization occurs due to the chronicity of the disease, following the Anna Karenina principle (AKP).

We also noted an attenuation of virulence-related functionalities and stress response from LAP to GAP to CP as evidenced by 4- to 144-fold lower levels of gram-negative cell wall components (including LPS and Lipid A) and fermentation, and of genes in the glutathione pathway in GAP when compared to LAP and in CP when compared to GAP. This serves to explain how the microbiome mediates the differential periodontal destruction observed in LAP, GAP, and CP. Virulence genes play an important role in the creation of a dysbiotic ecosystem, since they allow the species expressing them to overcome the colonization resistance offered by the health-compatible indigenous species. However, virulence comes at an extreme fitness cost, and therefore, bacteria benefit by staying avirulent unless environmental cues or competition dictate it. Our data suggests that attenuation of virulence potential might be a factor contributing to chronicity of the disease. This might explain the random burst model of disease progression [60], where an increase in community virulence precedes bursts of inflammation and loss of tooth-supporting structures.

## Conclusions

In summary, a comprehensive metagenomic analysis of the subgingival microbiomes in different disease phenotypes reveals broad patterns of shift in microbial functions that span all diseases. Many of these functions facilitate life in an oxygen-poor, protein- and heme-rich, pro-oxidant environment, as well as providing an enhanced capacity for attachment and biofilm formation. However, beta-dispersion metrics demonstrate that no two individuals with disease are alike, especially older individuals with chronic disease phenotype. Therapies focused on microbial modulation through mechanical, chemical, or other means will have to take into account patient-specific parameters for efficacy. Importantly, we observe that generalized aggressive periodontitis shares significant functional features with both localized aggressive periodontitis and chronic periodontitis, suggesting either attenuation of an aggressive disease or an early-onset chronic disease. We therefore question whether this is a separate disease entity, or an artifact induced by cross-sectional study designs. The present investigation also uncovers disease-specific indicators with varying discriminant abilities for each phenotype. These can not only serve as potential biomarkers for molecular identification of clinical phenotypes, but also clarify the role of the microbiome in disease pathogenesis.

## Supplementary Information

**Additional file 1: Supplemental Table 1.** Genus and species-level taxa that differed between chronic, localized and generalized aggressive periodontitis are shown. Differences in detection frequency (Fisher's exact test), differential abundance (DESeq2 along with log (2) fold differences, p-value, and FDR adjusted p-value (Wald’s Test), drivers of the class separation (SIMPER), species that were identified in ≥ 80% of individuals (common core microbiome) are shown.

**Additional file 2: Supplemental Table 2.** Same players, different teams. Network statistics of co-occurrence plots are shown.

**Additional file 3: Supplemental Table 3.** Periodontitis and the Anna Karenina principle. Genes that differed between chronic, localized and generalized aggressive periodontitis are shown, along with log (2) fold differences, p-value, and FDR adjusted p-value (Wald’s Test).

**Additional file 4: Supplemental Table 4.** GAP-a microbial chimera. Genes that were shared by all three diseases, as well as between generalized and localized aggressive periodontitis and generalized aggressive periodontitis and chronic periodontitis.

## Data Availability

Sequences for all 59 samples are deposited in the Sequence Read Archives under the project ID PRJNA552294 and PRJNA508385.
